# Conventional CT versus Dedicated CT Angiography in DIEP Flap Planning: A Feasibility Study

**DOI:** 10.3390/jpm11040277

**Published:** 2021-04-07

**Authors:** Anna D’Angelo, Alessandro Cina, Giulia Macrì, Paolo Belli, Sara Mercogliano, Pierluigi Barbieri, Cristina Grippo, Gianluca Franceschini, Sabatino D’Archi, Elena Jane Mason, Giuseppe Visconti, Liliana Barone Adesi, Marzia Salgarello, Riccardo Manfredi

**Affiliations:** 1 Dipartimento di Diagnostica per Immagini, Radioterapia Oncologica ed Ematologia, Fondazione Policlinico Universitario Agostino Gemelli IRCCS, 00168 Rome, Italy; alessandro.cina@policlinicogemelli.it (A.C.); paolo.belli@policlinicogemelli.it (P.B.); saramercogliano90@gmail.com (S.M.); pierluigi.barb@gmail.com (P.B.); riccardo.manfredi@policlinicogemelli.it (R.M.); 2 Divisione di Chirurgia Plastica, Dipartimento di Scienze della Salute della Donna e del Bambino e di Sanità Pubblica, Fondazione Policlinico Universitario Agostino Gemelli IRCCS, 00168 Rome, Italy; giulia.macri@live.com (G.M.); joevisconti@hotmail.com (G.V.); lbaroneadesi@libero.it (L.B.A.); marzia.salgarello@gmail.com (M.S.); 3 Dipartimento di Diagnostica per Immagini, Radiologia Terapeutica ed Interventistica, Azienda Ospedaliera Santa Maria Terni, 05100 Terni, Italy; cris.grippo@gmail.com; 4 Centro Integrato di Senologia, Dipartimento di Scienze della Salute della Donna e del Bambino e di Sanità Pubblica, Fondazione Policlinico Universitario Agostino Gemelli IRCCS, 00168 Rome, Italy; gianlucafranceschini70@gmail.com (G.F.); sabatinodarchi@gmail.com (S.D.); elenajanemason@gmail.com (E.J.M.)

**Keywords:** breast cancer, conventional CT and CT angiography, DIEP flap planning

## Abstract

The deep inferior epigastric perforator (DIEP) flap is used with increasing frequency in post-mastectomy breast reconstruction. Preoperative mapping with CT angiography (CTa) is crucial in reducing surgical complications and optimizing surgical techniques. Our study’s goal was to investigate the accuracy of conventional CT (cCT), performed during disease staging, compared to CTa in preoperative DIEP flap planning. In this retrospective, single-center study, we enrolled patients scheduled for mastectomy and DIEP flap breast reconstruction, subjected to cCT within 24 months after CTa. We included 35 patients in the study. cCT accuracy was 95% (CI 0.80–0.98) in assessing the three largest perforators, 100% (CI 0.89–100) in assessing the dominant perforator, 93% (CI 0.71–0.94) in assessing the perforator intramuscular course, and 90.6% (CI 0.79–0.98) in assessing superficial venous communications. Superficial inferior epigastric artery (SIEA) caliber was recognized in 90% of cases (CI 0.84–0.99), with an excellent assessment of superficial inferior epigastric vein (SIEV) integrity (96% of cases, CI 0.84–0.99), and a lower accuracy in the evaluation of deep inferior epigastric artery (DIEA) branching type (85% of cases, CI 0.69–0.93). The mean X-ray dose spared would have been 788 ± 255 mGy/cm. Our study shows that cCT is as accurate as CTa in DIEP flap surgery planning.

## 1. Introduction

**Figure 1:**
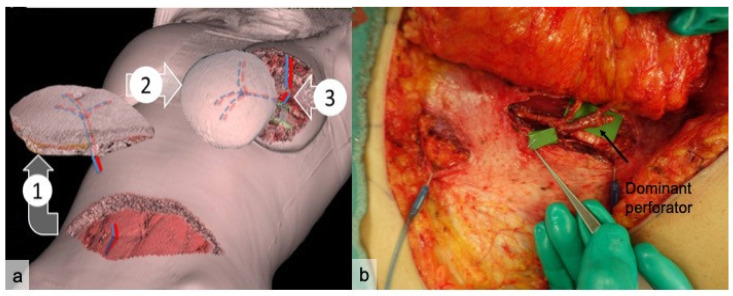
Three-dimensional graphic illustration of a DIEP (deep inferior epigastric perforator) flap procedure (**a**). In 1, skin and fat, with the perforating vascular pedicle from the deep inferior epigastric artery, are dissected from the abdominal wall; in 2 the flap is sized to reconstruct the breast; in 3 the internal mammary vessels are anastomosed to the vascular pedicle of the flap. (**b**) A surgical view of a DIEP dissection. The rectus abdominis is dissected with its fascia to isolate the inferior epigastric pedicle with its dominant perforator (arrow). Microgrid was employed to measure perforator caliber.

**Figure 2:**
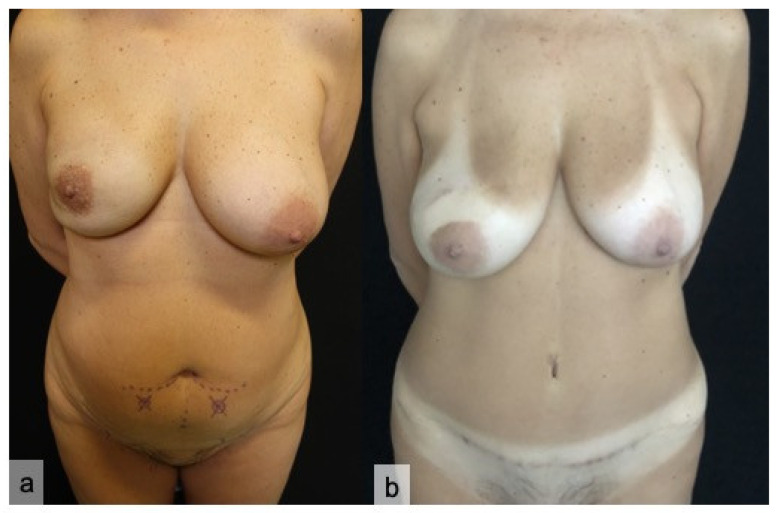
Preoperative planning (**a**) of a DIEP flap reconstruction for right breast carcinoma, requiring nipple-sparing mastectomy. Eight-month postoperative result (**b**).

The deep inferior epigastric perforator (DIEP) flap is, nowadays, considered the “gold standard” in autologous breast reconstruction [1]. Subcutaneous tissue and skin are transferred from the abdomen to the thorax in order to guarantee a more natural appearance of the reconstructed breast, compared to heterologous approach [2,3] (Figure 1 and Figure 2). A low donor site morbidity with an aesthetical abdomen improvement is an important factor for choosing DIEP flap in autologous breast reconstruction. The inconsistent anatomy of the abdominal perforators leads to a more challenging and time-consuming technique compared to a (muscle sparing) Transverse Rectus Abdominis Muscle (TRAM) flap [4,5].

Preoperative planning is crucial [6] in order to identify perforator vessels originating from the deep inferior epigastric vascular system, and to evaluate superficial inferior epigastric vessels. DIEP flap survival depends on adequate blood supply, which is guaranteed by perforator vessels that are amply variable in terms of number, anatomical location, intramuscular course, caliber, and tortuosity. Preoperative assessment includes visualization of the deep inferior epigastric artery (DIEA) and evaluation of its intramuscular course and branching pattern. The latter is described by Taylor’s classification, which defines three types of DIEA branching above the arcuate line: in type I the artery ascends as a single intramuscular vessel; in type II, the artery divides, at the arcuate line, into two vessels with an intramuscular course; in type III, the artery divides, at the arcuate line, into three vessels with an intramuscular course [7].

The DIEA originates from the external iliac artery, above the inguinal ligament, and crosses the lateral margin of the rectus abdominis muscle 3–4 cm below the arcuate line, with an average pedicle length of 10.3 cm and an average vessel diameter of 3.6 mm [8]. It then normally divides into two branches, lateral and medial; in case of a central course (28%), multiple small branches with centrally located perforators can be detected [9].

Perforators arise on each side of the midline from the anterior rectus fascia in a central rectangular area, which extends craniocaudally from 2 cm above to 6 cm below the umbilicus, and laterally between 1 cm and 6 cm from the midline. A thorough preoperative anatomical study also allows an assessment of the communications between the superficial and deep systems. The caliber of the superficial inferior epigastric artery (SIEA) should be compared to that of the dominant perforator, in order to select the best pedicle for the flap. In addition, assessing the integrity of the superficial inferior epigastric veins (SIEVs) could be helpful, in case of a flap additional venous discharge requirement [8].

Different perforator locations are associated with a harder or easier dissection, and sometimes lead to extensive splitting of the muscle; compared to lateral vessels, medial perforators offer better flap perfusion but a harder dissection due to a long intramuscular course. Perforator dissection is carried out along the deep inferior epigastric pedicle up to its origin from the external iliac artery. The DIEP flap should be adapted and shaped to the single patient and type of breast reconstruction, with an optimized anatomical preoperative study that allows the identification of personal anatomical characteristics in order to accelerate dissection and flap harvesting, as well as to avoid vascularization deficiencies. An accurate preoperative planning with evaluation of single anatomical variants allows a decrease in decrease operating time and theatre utilization, with a consequent benefit in terms of surgical waiting lists and staff optimization.

Among the available imaging techniques, which include Magnetic Resonance Imaging (MRI) and color-Doppler ultrasound (US) [3,10], Computed Tomography Angiography (CTa), with the injection of contrast medium, has become the gold standard in planning surgery [11,12] thanks to its ability to map out the vascular anatomy and, consequently, select the best DIEP flap to harvest. Its high accuracy has been proved in studies performed, both on cadavers [8] and post-surgery. CTa also allows 3D surface and vascular tree-rendering [9], which can bring huge benefits to cross-sectional imaging and represents a valid visual tool for surgeons. The primary role of CTa in preoperative assessment is, therefore, motivated by its wide availability, fast acquisition time, high reproducibility, and great sensitivity in the identification of perforator vessels with calibers larger than 1mm. Still, CTa is associated with possible complications, such as allergic reactions to contrast medium, nephrotoxicity in patients with impaired renal function, and exposure to ionizing radiation in patients often already subjected to multiple CT scans to stage primary breast cancer [13].

Our goal was to investigate the accuracy of conventional CT (cCT), performed during breast cancer disease staging, compared to CTa in obtaining information required for DIEP flap surgical planning. We evaluated the accuracy of both techniques in identifying “dominant” perforator arteries, measuring their caliber and intramuscular course length, assessing superficial venous communications (SVC) and DIEA branching type according to Taylor’s classification, identifying the caliber of SIEA, and assessing SIEV integrity. In addition, the total X-ray dose that could have been potentially spared by avoiding CTa was evaluated.

## 2. Materials and Methods

From January 2010 to February 2019, 344 patients programmed to receive mastectomies with immediate or delayed DIEP flap reconstruction, referred to our Institute, were enrolled in the study. Inclusion criteria were: cCT performed during disease staging with standardized technique (slice thickness of 1.25 mm in the portal venous-phase) in our Institution within 24 months after CTa. Exclusion criteria were: abdominal surgery between the two examinations or cCT performed in other Institutions.

This retrospective single-center study was conducted according to the guidelines of the Declaration of Helsinki, and approved by the Institutional Review Board and Ethics Committee of Fondazione Policlinico Universitario Agostino Gemelli IRCCS on 11 June 2020. Anyone involved in the research agreed to participate and agreed to have the results of the research about them published.

### 2.1. CTa and cCT Technique

CTa and cCT were performed using a 64-slice multidetector CT (LightSpeed VCT, GE Healthcare, Waukeska, WI, USA), table travel per rotation was 23 mm (gantry rotation time 0.4 s) and field of view (FOV) was 40 cm in order to match patient width, matrix side 512 × 512. Tube voltage was 120 kVp, with Smart mAs (GE Healthcare) dose enabled (noise index set to 22). For CTa, the arterial-phase images were acquired at a 0.65 mm slice thickness; to minimize radiation exposure, a small field of view (FOV), which only includes the area of interest, is scanned: from the origin of the inferior epigastric artery at the level of the groin to a level approximately 3 cm above the umbilicus in a caudal-cranial direction. We administered, intravenously, 100 mL of iodinated contrast medium (Ultravist, Bayer Schering Pharma AG, Berlin, Germany) with a concentration of 370 mgL/mL (18-G cannula) at 4 mL/s flow rate, followed by 60 mL saline flush. A large-gauge (18 G) peripheral intravenous line was preferred to allow rapid infusion of contrast (4–5 mL/s) and, thus, an optimal opacification of small epigastric vessels. The arterial peak of enhancement was captured using bolus tracking (Smart Prep, GE Healthcare, Wuakuesha, WI, USA), so as to begin image acquisition upon the contrast medium arrival in the region of interest (ROI) on the common femoral artery; acquisition should be obtained with a minimum possible delay after contrast arrival is detected, with blood attenuation within ROI of 100–120 Hounsfield units (HU). During the exam, since a whole scan can be accomplished in one held breath, and the effect of breathing motion on the abdomen and pelvis may be relevant, patients are required to hold their breath and are supine, with their arms placed according to the programmed sugery (upwards for immediate breast reconstruction, downwards in case of delayed reconstruction).

For cCT, the venous-phase images were acquired at a 1.25 mm slice thickness, in cranio-caudal direction, with patients in a supine position with their arms lying upwards. Following our department’s routine for oncologic staging cCT, 1.6 mL/kg of contrast medium (Ultravist 370 mgL/mL) was administered to patients at a rate of 3 mL/s, followed by 40 mL of saline solution at the same injection rate. The scan delay was empirically chosen at 70 s.

### 2.2. Image Analysis

**Figure 3:**
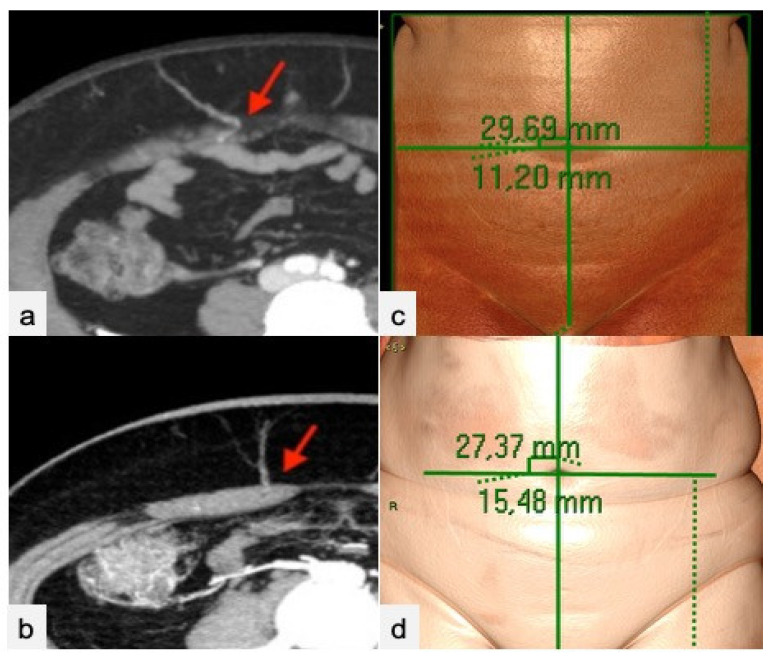
Dominant perforator’s emergence from the anterior rectus abdominis fascia (red arrows) in cCT (**a**) and CTa (**b**) axial sub-volume maximum intensity projection (MIP) reconstructions. Images (**c**,**d**) show mapping of the dominant perforator on a VR reconstruction of the abdominal surface via a virtual coordinate system centered on a zero point, corresponding to the umbilicus in cCT (**c**) and CTa (**d**).

**Figure 4:**
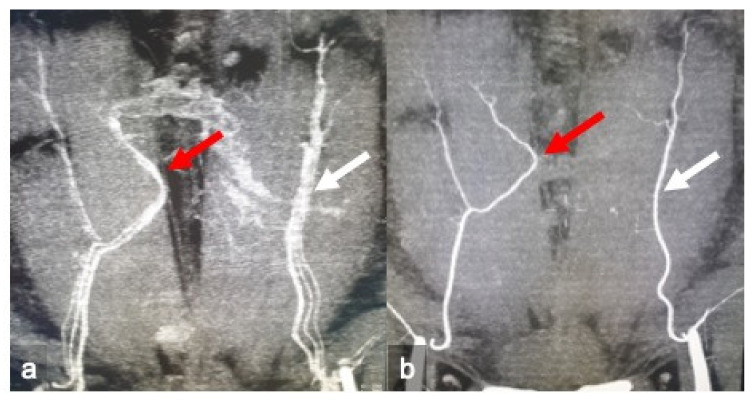
Identification of the deep inferior epigastric artery branching according to Taylor’s classification. cCT (**a**) and CTa (**b**) oblique-coronal sub-volume maximum intensity projection reconstruction (MIP) of the superficial abdominal wall revealed a bifurcated artery on the right hemi-abdomen (red arrows) and a single on the left (white arrows).

**Figure 5:**
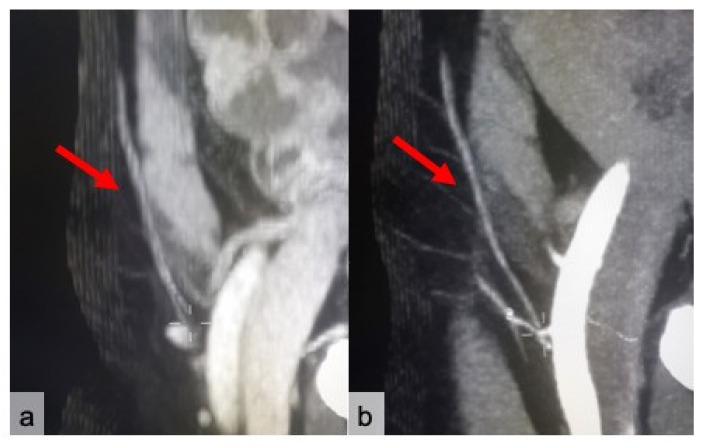
Assessment of the SIEA caliber compared to the dominant perforator. cCT (**a**) and CTa (**b**) sub-volume sagittal MIP reconstructions show a SIEA (red arrows) with a 2 score (equal to the dominant perforator).

**Figure 6:**
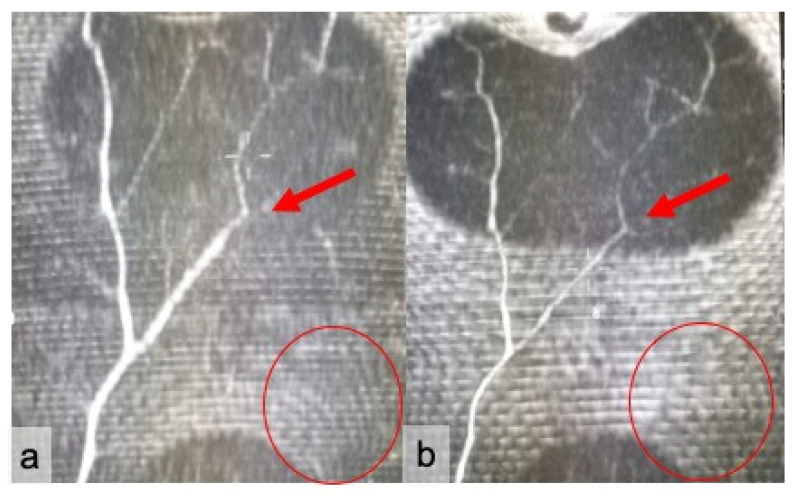
Assessment of superficial venous communications running between the right and left portion of the abdomen. Coronal sub-volume maximum intensity projection (MIP) reconstructions of the superficial abdominal wall for cCT (**a**) and CTa (**b**) show a large venous trunk on the right hemi-abdomen (red arrows), with a 3 score. Superficial inferior epigastric vein integrity was absent on the left (red circles).

Two radiologists with specific experience in flap surgery imaging reviewed, respectively, the cCT and aCT exams to assess the diagnostic accuracy of cCT in identifying: the main perforators, the “dominant” perforator, and the perforation site of the rectus abdominal fascia using volumetric reconstructions. The errors on x and y virtual coordinates were then calculated (Figure 3). The reader also evaluated the course of the dominant perforator, assigning a value from 1 to 4 (“1” extramuscular, “2” intramuscular for a length <2 cm, “3” <4 cm and “4” >4 cm); the branching of the DIEA according to Taylor’s classification (Figure 4); the caliber of the SIEA compared to the dominant perforator (from 1 to 3, “1” <dominant, “2” =dominant, “3” >dominant) (Figure 5); the integrity of the SIEV (from 1 to 3, “1” intact, “2” attracted, “3” interrupted); and the presence of superficial venous communications between the right and left hemi-abdomen (“0” if absent, “1” scarce, “2” moderate, “3” clearly evident) (Figure 6).

### 2.3. Statistical Analysis

A Shapiro–Wilk test was employed to assess the parametric vs nonparametric distribution of variables. Continuous variables were described by mean and standard deviation. The accuracy of cCT was tested, with CTa employed as a standard of reference. Confidence intervals were reported at 95%. For inferential statistics, a Student *t*-test and Wilcoxon rank–sum test were employed, respectively, for parametric and nonparametric variables. Setting a type II error (1 − β) of 0.9 and a Type I error rate of 0.05, and assuming as clinically relevant a 0.9 accuracy of the cCT vs. CTa, a sample size of 35 patients was needed.

## 3. Results

We enrolled 35 patients with a mean age of 40 years (range 27–73 years) and a mean BMI of 25,2 kg/m^2^ (range 21.2–32.3). No statistically significant differences were observed in patient characteristics. The accuracy of cCT in assessing the three largest perforators was 95% (CI 0.80–0.98). The dominant perforator was identified by cCT in all cases (100%, CI 0.89–100). cCT correctly identified the perforator intramuscular course in 93% of cases (CI 0.71–0.94) and the superficial venous communications in 90.6% of patients (CI 0.79–0.98). The SIEA caliber was correctly assessed by cCT in 90% of cases (CI 0.84–0.99). cCT was less accurate in the evaluation of DIEA branching type (85% of cases, CI 0.69–0.93), but had an excellent assessment of the integrity of SIEV (96% of cases, CI 0.84–0.99). The mean error in topographic localization was 4.8 ± 3.8 mm along the Y axis and 2.6 ± 3.8 mm along the X axis. If CTa had been spared before surgery, relying on cCT for DIEP planning, the mean X-ray dose potentially avoided would have been 788 ± 255 mGy/cm. Data reported are shown in Table 1.

## 4. Discussion

The results of our study show that cCT, performed routinely during breast cancer disease staging, is as accurate as CTa in obtaining information required for DIEP flap planning. CTa, first described by Rozen in 2008 [8], has been suggested as the gold standard in preoperative assessment of perforating vessels. Other modalities, such as MRI [14] and color-Doppler US, have been compared to CTa. Preoperative breast MRI performed for breast malignancy characterization can be extended to the lower abdomen, but still allows visualization and localization of only some of the perforator vessels, as it possesses a lower spatial resolution compared to CT angiography [15]. The prone position required to perform breast MRI modifies the natural anatomy of the abdomen, and, together with artefacts due to respiratory movements and enhanced vascular assessment, constitutes a limitation to using MRI, as reported in our previous study [11]. Color-Doppler US, though it offers a more accurate spatial resolution than CTa, is an operator dependent procedure and requires advanced training to obtain a satisfying mapping of perforators [16]. To our knowledge, no previous studies investigated the role of CTa versus cCT. Our results show an excellent diagnostic accuracy of cCT in identifying the three largest perforators, the perforator intramuscular course, SCVs, the dominant perforator, SIEA caliber, and SIEV integrity. cCT was less accurate in the evaluation of DIEA branching type, probably because of lower contrast resolution during the venous phase, different contrast medium injection speed, and the cranial-caudal direction of acquisition. The mean error in topographic localization of the dominant perforator was 4.8 ± 3.8 mm along the Y axis and 2.6 ± 3.8 mm along the X axis, probably because of the different arm position in delayed surgical reconstruction and the presence of clothes (knickers) when the cCT is performed. Results from both techniques were compared with intraoperative findings: all preoperatively assessed dominant perforators were confirmed intraoperatively, without significant differences in terms of expected position.

Our study suggests that performing cCT alone, in the preoperative assessment of DIEP-flap candidates, is safe and feasible. Furthermore, everyday clinical practice could benefit from the adoption of this technique in several ways: preoperative assessment is faster without the necessity of programming a CTa exam; there is also the matter of reduced healthcare costs and patient discomfort, both in terms of psychological stress and x-ray or contrast medium exposure. This evidence is particularly significant when dealing with patients who are already exposed, because of their underlying disease, to multiple CT examinations. If CTa had been withheld before surgery, relying on cCT alone for DIEP planning, the patient would have been spared a mean X-ray dose of 788 ± 255 mGy/cm. Furthermore, this technique is easily applicable to most centers around the world, including facilities without access to CTa, as it does not require a dedicated acquisition protocol or a radiologist specialized in vascular anatomy.

Our study has some limitations, the major of which being that we could not assess interobserver variability between CTa and cCT because only one experienced radiologist was present for each method. Furthermore, DIEP flap procedure total surgical time was not taken into account in this study, although it was widely analyzed in our previous manuscript [3].

## 5. Conclusions

We found that cCT, although not intentionally performed for preoperative surgical assessment, nonetheless provided an accurate visualization of the best perforator and of the main abdominal vessels involved in DIEP planning, thus, overcoming the limits of US in terms of reproducibility and operator dependence, and of MRI in terms of spatial resolution, costs, and artifacts related to the prone position. In this way, patients scheduled for DIEP flap surgery with a recent cCT could avoid further assessment with CTa. In conclusion, in order to strongly reduce radiation exposure, time, and costs in DIEP flap planning, a previous recent cCT may be a valuable option due to high concordance with CTa findings.

## Figures and Tables

**Table 1:** Performance of cCT versus CTa.

Items	%	CI
3 largest perforators	95%	0.80–0.98
Dominant perforator	100%	0.89–100
Perforator intramuscular course	93%	0.71–0.94
Superficial venous communications	90.6%	0.79–0.98
DIEA branching type	85%	0.69–0.93
SIEA calibre	90%	0.84–0.99
Integrity of SIEV	96%	0.84–0.99

## Data Availability

The data presented in the study are available on request from the corresponding author.

## References

[1] Munhoz A.M., Arruda E., Montag E., Aldrighi C., Aldrighi J.M., Gemperli R., Ferreira M.C. (2007). Immediate skin-sparing mastectomy reconstruction with deep inferior epigastric perforator (DIEP) flap: Technical aspects and outcome. Breast J..

[2] Ireton J.E., Lakhiani C., Saint-Cyr M. (2014). Vascular anatomy of the deep inferior epigastric artery perforator flap: A systematic review. Plast. Reconstr. Surg..

[3] Cina A., Salgarello M., Barone-Adesi L., Rinaldi P., Bonomo L. (2010). Planning breast reconstruction with deep inferior epigastric artery perforating vessels: Multidetector CT angiography versus color Doppler US. Radiology.

[4] Allen R.J. (2001). Comparison of the costs of DIEP and TRAM flaps. Plast. Reconstr. Surg..

[5] Kaplan J.L., Allen R.J. (2000). Cost-based comparison between perforator flaps and TRAM flaps for breast reconstruction. Plast. Reconstr. Surg..

[6] Tønseth K.A., Hokland B.M., Tindholdt T.T., Abyholm F.E., Stavem K. (2008). Quality of life, patient satisfaction and cosmetic outcome after breast reconstruction using DIEP flap or expandable breast implant. J. Plast. Reconstr. Aesthet. Surg..

[7] Taylor G.I., Hamdy H., El-Mrakby H.H., Milner R.H. (2002). Vascular anatomy of the lower anterior abdominal wall: A microdissection study on the deep inferior epigastric vessels and the perforator branches. Plast. Reconstr. Surg..

[8] Rozen W.M., Ashton M.W., Stella D.L., Phillips T.J., Taylor G.I. (2008). The accuracy of computed tomographic angiography for mapping the perforators of the DIEA: A cadaveric study. Plast. Reconstr. Surg..

[9] Gacto-Sánchez P., Sicilia-Castro D., Gómez-Cía T., Lagares A., Collell T., Suárez C., Parra C., Infante-Cossío P., De La Higuera J.M. (2010). Use of a Three-Dimensional Virtual Reality Model for Preoperative Imaging in DIEP Flap Breast Reconstruction. J. Surg. Res..

[10] Giunta R.E., Geisweid A., Feller A.M. (2000). The value of preoperative doppler sonography for planning free perforator flaps. Plast. Reconstr. Surg..

[11] Cina A., Barone-Adesi L., Rinaldi P., Cipriani A., Salgarello M., Masetti R., Bonomo L. (2013). Planning deep inferior epigastric perforator flaps for breast reconstruction: A comparison between multidetector computed tomography and magnetic resonance angiography. Eur. Radiol..

[12] Casey W.J., Chew R.T., Rebecca A.M., Smith A.A., Collins J.M., Pockaj B.A. (2009). Advantages of preoperative computed tomography in deep inferior epigastric artery perforator flap breast reconstruction. Plast. Reconstr. Surg..

[13] McMillan K., Bostani M., Cagnon C.H., Yu L., Leng S., McCollough C.H., McNitt-Gray M.F. (2017). Estimating patient dose from CT exams that use automatic exposure control: Development and validation of methods to accurately estimate tube current values. Med. Phys..

[14] Schaverien M., Ludman C., Neil-Dwyer J., Perks G.B., Akhtar N., Rodrigues J.N., Benetatos K., Raurell A., Rasheed T., McCulley S.J. (2011). Contrast-Enhanced magnetic resonance angiography for preoperative imaging in DIEP flap breast reconstruction. Plast. Reconstr. Surg..

[15] Rozen W.M., Stella D.L., Bowden J., Taylor G.I., Ashton M.W. (2009). Advances in the pre-operative planning of deep inferior epigastric artery perforator flaps: Magnetic resonance angiography. Microsurgery.

[16] Klasson S., Svensson H., Malm K., Wassélius J., Velander P. (2015). Preoperative CT angiography versus Doppler ultrasound mapping of abdominal perforator in DIEP breast reconstructions: A randomized prospective study. J. Plast. Reconstr. Aesthet. Surg..

